# Fabry disease screening in high-risk populations in Japan: a nationwide study

**DOI:** 10.1186/s13023-020-01494-6

**Published:** 2020-08-26

**Authors:** Shinichiro Yoshida, Jun Kido, Takaaki Sawada, Ken Momosaki, Keishin Sugawara, Shirou Matsumoto, Fumio Endo, Kimitoshi Nakamura

**Affiliations:** 1 Department of Pediatrics, Graduate School of Medical Sciences, Kumamoto University, 1-1-1 Honjo, Chuo-ku, Kumamoto City, Kumamoto, 860-8556 Japan; 2 KM Biologics Co., Ltd., Kumamoto, Japan; 3 Kumamoto-Ezuko Medical Center for Disabled Children, Kumamoto, Japan

**Keywords:** Fabry disease, High-risk screening, α-Galactosidase a, *GLA* gene, Novel variants

## Abstract

**Background:**

Fabry disease (FD) is a X-linked inherited disorder caused by mutations in the *GLA* gene, which results in the deficiency of α-galactosidase A (α-Gal A). This leads to the progressive accumulation of metabolites, which can cause multisystemic dysfunction. A recent screening study among neonates reported an increase in the incidence of FD, and numerous FD patients remain undiagnosed or even misdiagnosed. Therefore, this study aimed to identify patients with FD by performing high-risk screening in 18,135 individuals, enrolled from October 2006 to March 2019, with renal, cardiac, or neurological manifestations from all prefectures in Japan. A total of 601 hospitals participated in this study.

**Results:**

Low α-Gal A activity was detected in 846 individuals, with 224 of them diagnosed with FD by *GLA* sequencing. Cases with a family history of FD (*n* = 64) were also subjected to sequencing, without α-Gal A assay, as per individual request, and 12 of them were diagnosed with a variant of FD. A total of 236 patients with FD (97 males and 139 females) were identified from among 18,199 participants. A total of 101 *GLA* variants, including 26 novel variants, were detected in the 236 patients with FD from 143 families, with 39 amenable variants (39%) and 79 of the 236 patients (33%) suitable for migalastat treatment.

**Conclusions:**

From among 18,199 participants, 101 *GLA* variants, including 26 novel variants, were identified in the 236 patients with FD from 143 families. Migalastat was identified as a suitable treatment option in 33% of the patients with FD and 39% of the *GLA* variants were detected as amenable. Therefore, the simple screening protocol using dried blood spots that was performed in this study could be useful for early diagnosis and selection of appropriate treatments for FD in high-risk and underdiagnosed patients with various renal, cardiac, or neurological manifestations.

## Background

Fabry disease (FD; OMIM 301500) is an inherited X-linked disorder caused by mutations in the *GLA* gene, which encodes the lysosomal enzyme α-galactosidase A (α-Gal A; EC 3.2.1.22). To date, 516 and 612 *GLA* variants have been incorporated into the Fabry-database (Fabry-database.org, ver. 3.2.2, last updated on February 15, 2019) [1] and ClinVar (http://www.ncbi.nlm.nih.gov/clinvar) [2], respectively. The functional deficiency of α-Gal A results in the progressive accumulation of metabolites, such as globotriaosylceramide in lysosomes, biological fluids, and the vascular endothelium, which can cause disease manifestations in the skin, eyes, kidneys, ears, lungs, heart, and brain [3–5]. FD patients who have very low α-Gal A activity exhibit the classic phenotype and are generally asymptomatic in early childhood [6, 7]. In contrast, FD patients with residual α-Gal A activity exhibit milder clinical manifestations and onset occurs later than in those with the classic phenotype. Heterozygous women with pathogenic *GLA* variants are not only carriers but also express wide manifestation spectra, ranging from asymptomatic to as severe as those of the classic phenotype, depending on random X-chromosomal inactivation [8].

Clinical manifestations are multisystemic, including limb pain, acroparesthesia, angiokeratoma, anhidrosis, and corneal opacity in childhood, with a progression to major organ involvement in adulthood, such as proteinuria, impaired renal function, cardiomyopathy, and stroke. Because these manifestations are frequently observed in individuals with diabetes, hypertension, and arteriosclerosis, which are nonspecific, this might lead to a delayed or an incorrect diagnosis [9, 10]. Recent newborn screening (NBS) studies, including our previous study [11], reported that the incidence of FD was as high as 1:1600 to 1:8485 in live births [12, 13]. Therefore, the prevalence of FD is underestimated, with evidence suggesting that there are many undiagnosed or misdiagnosed FD patients.

Enzyme replacement therapy (ERT) is now available in Japan and three products are on the market, namely Fabrazyme® (Sanofi Genzyme), Replagal® (Shire), Agalsidase beta BS (JCR). Moreover, an oral pharmacological chaperone, migalastat (Galafold®; Amicus Therapeutics), has become available for specific pathogenic *GLA* variants, i.e., migalastat-amenable *GLA* variants [14]. ERT can decelerate renal deterioration and the progression of cardiomyopathy, thereby delaying morbidity and mortality [15]. Migalastat has the same effects on renal function as ERT [16]. Early treatment is essential to preserve organ function and prevent progression of the disease. The high-risk screening for FD is considered a practical strategy for early treatment.

Nakagawa et al. [17] reported partial results regarding high-risk Japanese patients in the Hokkaido prefecture with cardiac, renal, or neurological manifestations. This study aimed to identify undiagnosed patients with FD by performing high-risk screening among 18,199 individuals with renal, cardiac, or neurological manifestations, from all of the prefectures in Japan, and to assess the effectiveness of our simple screening protocol for the definite diagnosis of FD in the aforementioned high-risk groups.

## Results

### High-risk screening for Fabry disease

**Fig. 1:**
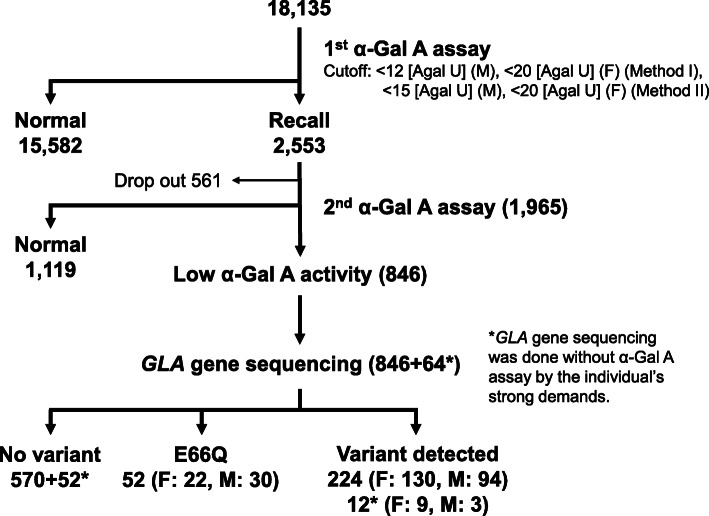
Schematic representation of high-risk screening for Fabry disease

**Fig. 2:**
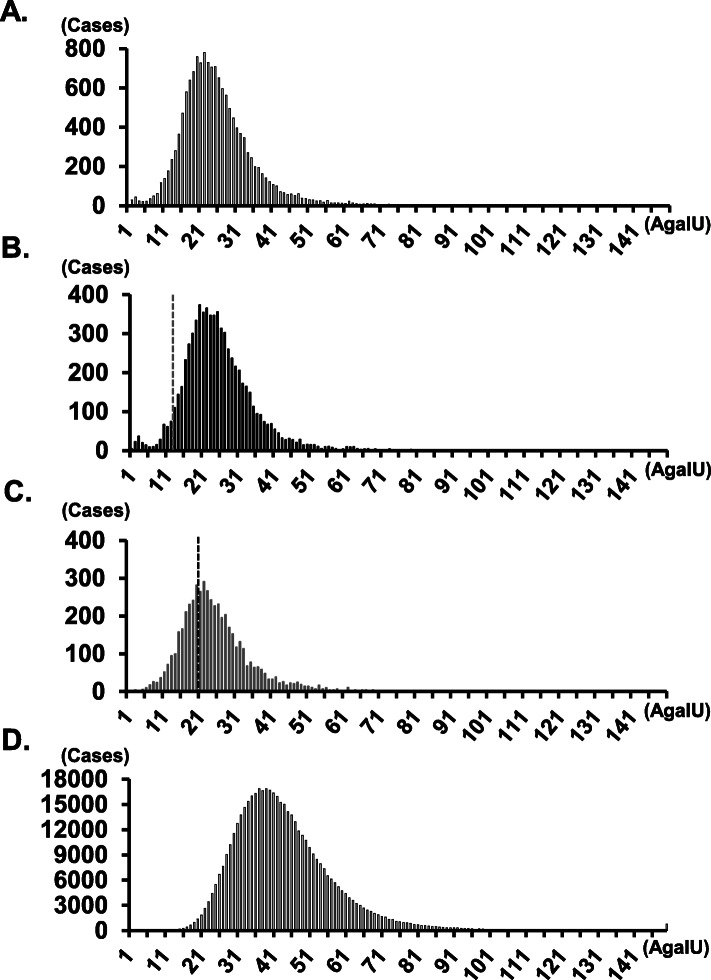
Histograms of α-Gal A activity in the high-risk and newborn screening groups. Histograms of α-Gal A activity are shown for (**a**) the total population (*N* = 16,061), (**b**) men (*N* = 10,066), and (**c**) women (*N* = 5558) in the high-risk screening group, as well as (**d**) the newborn screening group (*N* = 483,026). Dashed line: cutoff level

The demographic characteristics of the enrolled individuals are summarized in Table 1. Of the 18,135 individuals that were screened, 8823 had renal manifestations (5505 males, 3090 females, and 228 did not provide information regarding sex), 4057 presented signs of cardiac manifestations (2853 males, 1176 females; 28 did not provide information regarding sex), 3075 evidenced central neurological manifestations (1926 males, 968 females; 181 did not provide information regarding sex), 894 experienced peripheral neurological manifestations (522 males, 371 females; 1 did not provide information regarding sex), 715 had a family history of FD (333 males, 378 females; 4 did not provide information regarding sex), and 571 were classified as “other” (350 males, 206 females; 15 did not provide information regarding sex). The flow chart and results of the high-risk screening program for FD are presented in Fig. 1. The 2553 individuals, who had low α-Gal A activity, were recalled and 1965 of them proceeded to the second α-Gal A assay. Figures 2 and S1 shows the distribution of α-Gal A activity in the first assay in Method I. Approximately 4 and 28% of male and female patients, respectively, fell below the cutoff value. The median α-Gal A activity obtained using Method I was 24.47, 24.50, and 24.06 (AgalU) in among all individuals, men, and women, respectively (Fig. 2a, b, and c). The median α-Gal A activity obtained using Method II was 57.50, 62.54, and 45.53 (AgalU) among all individuals, men, and women, respectively. From Methods I and II, 846 individuals were identified and 224 of them were diagnosed with FD by *GLA* sequencing. No *GLA* gene variants were identified in 650 individuals, whereas 52 individuals presented with a possible functional polymorphism allele, p.E66Q. Of the 224 individuals that presented with *GLA* gene variants, 28 (19 male, 9 female), 29 (17 male, 12 female), 5 (3 male, 2 female), 30 (20 male, 10 female), 128 (32 male, 96 female), and 4 (3 male, 1 female) variants were identified in the renal, cardiac, central neurological, peripheral neurological, family history, and “other” groups, respectively (Table 1). The prevalence of FD was 0.42% (male: 0.45%, female: 0.38%), 0.94% (male: 0.77%, female: 1.33%), 0.22% (male: 0.20%, female: 0.27%), 4.37% (male: 4.98%, female: 3.50%), and 23.40% (male: 12.49%, female: 33.02%) in the renal, cardiac, central neurological, peripheral neurological, and family history groups, respectively. The median age was 49 years for men and 58 years for women, 47 years for men and 47.5 years for women, 13 years for both men and women, and 20 years for men and 38 years for women in the renal, cardiac, peripheral neurological, and family history groups, respectively. Individuals with a family history of FD (*n* = 64) were also subjected to sequencing analysis, without α-Gal A assay, as per individual request. *GLA* gene variants were detected in 12 (3 males and 9 females) of the 64 individuals. Therefore, a total of 236 FD patients were detected from 18,199 individuals in this high-risk screening study.

**Table 1:** Demographic characteristics of enrolled individuals

Group	Enrolled				Variant detected		
	Gender	n	Age (year)		n (%)$	Age (year)	
			Median	IQR		Median	IQR
a	M	5505	64	55–73	19 (0.45)	49	40–55
	F	3090	66	56–74	9 (0.38)	58	NC
	M or F	228					
b	M	2853	61	48–71	17 (0.77)	47	37–56
	F	1176	67	54–75	12 (1.33)	47.5	33.75–53.75
	M or F	28					
c	M	1926	67	54–77	3 (0.20)	NC	NC
	F	968	72	53–82	2 (0.27)	NC	NC
	M or F	181					
d	M	522	13	8–31	20 (4.98)	13	11–18.5
	F	371	14	9–41	10 (3.50)	13	9–25
	M or F	1					
e	M	333	28	11–45	32 (12.49)	20	13–39
	F	378	36	21–49	96 (33.02)	38	18.75–49.75
	M or F	4					
f	M	350	46	16–63	3 (1.11)	NC	NC
	F	206	43	14–64	1 (0.63)	NC	NC
	M or F	15					
	Total	18,135			224		

*IQR* Interquartile range (25–75%), *NC* Not calculable

$: The prevalence of FD was adjusted for the drop-out rate; e.g., 19/5505 × 2553/1965 = 0.45%

a: Renal manifestations group. b: Cardiac manifestations group. c: Central neurological manifestations group. d: Peripheral neurological manifestations group. e: Family history group. f: “Other” group

### *GLA* variants detected in the FD patients

Herein, 101 *GLA* variants were detected in 236 patients from 143 families (Table 2). Of these, 39 (39%) were amenable and 79 of the 236 patients were considered suitable for migalastat treatment (Table 2, Table S1 and Table S2). Regarding mutation type of the 101 variants, 64 were identified as missense mutations, 18 were frameshift mutations, 10 were nonsense mutations, 3 were in-frame deletions, 3 were intronic mutations, 2 were silent mutations (which potentially alter splicing), and 1 was a large deletion mutation of exon 3 and 4. Of the 101 variants, 68 were registered in ClinVar or Fabry-database.org. Two variants, c.218C > A [18] and c.908_928del21 [17], were described in our previous report which included detailed information on each patient. The variant c.625 T > C was detailed in our previous report regarding NBS for FD [11]. Four variants, namely c.725 T > C, c.801 + 1G > A, c.1124G > A, and c.1165C > G, were reported by Tsukimura et al. [19], Li et al. [20], Iwafuchi et al. [21], and van der Tol et al. [22], respectively. The remaining 26 variants were considered novel variants. The most common variant was c.888G > A/p.M296I (allele frequency: 3.5%, 5/143). The second most common variant was c.334C > T/p.R112C (2.8%, 4/143). The third most common variants were c.335G > A/p.R112H (2.1%, 3/143), c.658C > T/p.R220* (2.1%, 3/143), c.679C > T/p.R227* (2.1%, 3/143), c.718_719delAA/p.K240Efs*8 (2.1%, 3/143), c.902G > A/p.R301Q (2.1%, 3/143), c.1033_1034delTC/p.S345Rfs*28 (2.1%, 3/143), c.1124G > A/ p.G375E (2.1%, 3/143), and c.1235_1236delCT/p.T412Sfs*37 (2.1%, 3/143). The geographic distribution of the variants is illustrated in Fig. S2.

**Table 2:**
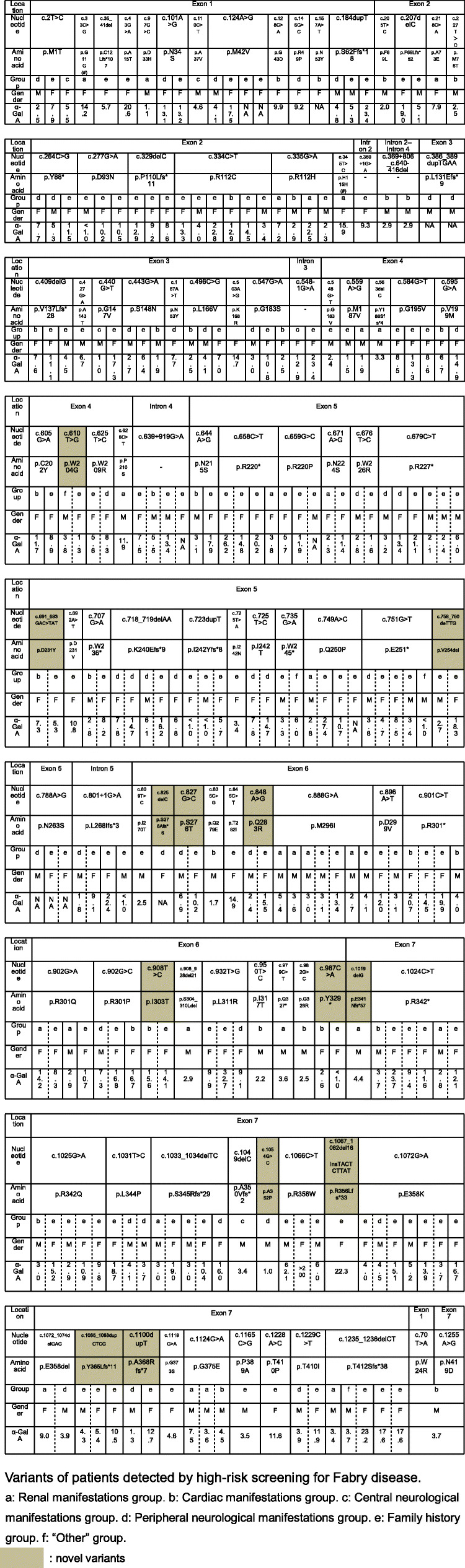
Variants of patients detected by high-risk screening for Fabry disease

## Discussion

High-risk screening for FD in selected patient cohorts has been reported. Doheny et al. [23] reanalyzed studies related to hemodialysis (27 reports, 23,954 men, and 12,866 women), left ventricular hypertrophy (LVH) and/or hypertrophic cardiomyopathy (17 reports, 4054 men, and 1437 women), and ischemic or cryptogenic strokes (16 studies, 3904 men, and 2074 women). The revised prevalence was estimated as 0.21% for male and 0.15% for female hemodialysis patients, 0.94% for male and 0.90% for female cardiac patients, and 0.13% for male and 0.14% for female stroke patients. In the current study, the prevalence was estimated as 0.42% (male: 0.45%, female: 0.38%) in the renal manifestation group (a), 0.94% (male: 0.77%, female: 1.33%) in the cardiac manifestation group (b), and 0.22% (male: 0.20%, female: 0.27%) in the central neurological manifestation group (c), which are comparable to those of previous reports.

The prevalence of FD in the peripheral neurological manifestation group (d) was second highest at 4.37% (male: 4.98%, female: 3.50%), whereas the patients’ age (male: median 13 [IQR: 11–18.5] years old, female: median 13 [IQR: 9–25] years old) was lesser than that of the other groups. Therefore, manifestations such as limb pain, acroparesthesia, clustered angiokeratoma, cornea verticillata, and hypo- or anhidrosis, could help identify FD patients. Politei et al. [24] has recommended that the cause of pain should be diagnosed early in unrecognized or newly diagnosed FD patients to improve treatment possibilities. FD experts consider that, regardless of sex or age, pain related to FD could be an early indication to commence ERT before potentially irreversible organ damage, to the kidneys, heart, or brain, prevails. However, a study conducted in Russia by Namazova-Baranova et al. [25] reported that no FD patients were identified from among 214 individuals (110 males and 104 females) with chronic limb pain. Moreover, the genetic, epidemiological, and ethnical information related to Russian FD patients are insufficient and future studies and information related to FD in Russia are required.

The prevalence of FD in individuals with a family history (e) was the highest at 23.40% (male: 12.49%, female: 33.02%). *GLA* sequencing for individuals with a family history of FD was useful in identifying undiagnosed or pre-symptomatic FD patients. Therefore, when patients experience FD-related symptoms, clinicians should confirm the presence of a family history of FD and, if applicable, whether similar symptoms developed.

The variant spectra of *GLA* in Japanese patients have been reported [26, 27]. *GLA* gene analysis was previously performed for 207 FD patients [26]. The most common variant was c.888G > A/p.M296I (allele frequency: 5.8%, 12/207). The second-most common variants were c.639 + 919G > A (4.3%, 9/207) and c.679C > T/p.R227* (4.3%, 9/207), followed by c.334C > T/p.R112C (3.9%, 8/207), c.335G > A/p.R112H (3.9%, 8/207), and c.902G > A/p.R301Q (3.9%, 8/207). In another study, 73 pathogenic variants were detected in 176 patients from 115 families [27] and the most common variant was c.334C > T/p.R112C (allele frequency: 2.65%). The second-most common variant was c.888G > A/p.M296I (1.89%), followed by c.658C > T/p.R220* (1.52%), c.718_719delAA/p.K240Efs*8 (1.52%), and c.1025G > A/p.R342Q (1.52%). The common variants identified in these studies, as well as those of the current study, overlapped.

We previously reported the first large-scale NBS program for FD in the western region of Japan [11]. A total of 599,711 newborns were screened and 26 *GLA* variants, including 8 novel variants, were detected in 57 neonates from 54 families. Of the 26 variants, 10 were also detected in the current study and most of them were detected in patients from western Japan (Fig. S1).

In the current study, 4 pedigrees (4/68, 5.9%) were perceived as de novo mutations (Table S3). The frequency might be comparable with those of previous studies performed in Japan (6.8% (5/74); Kobayashi et al. [28]), Italy (2.8% (3/108); Romani et al. [29]; and 14.3% (2/14); Morrone et al. [30]), Spain (4.5% (1/22); Rodriguez-Mari et al. [31]), and the United Kingdom (6.3% (1/16); Davies et al. [32]). A high frequency of de novo mutations has been reported in X-linked disorders, such as Duchenne muscular dystrophy (DMD) and hemophilia A (F8), and de novo mutations account for approximately one-third of the mutations in these two disorders [33, 34]. This is owing to lack of fitness to reproduce in the X chromosome. Hemizygous *GLA* mutations may have sufficient fitness to reproduce, reducing the frequency of de novo mutations [35]. Moreovrer, the presence of CpG dinucleotides also may increase mutational frequency [36]. *GLA* contains 19 CpG sites (1/68 bases) in the coding region compared to *F8* which has 68 CpG sites (1/104 bases). Of the 19 potential mutation sites in *GLA*, 13 variants were identified in the current study, namely, c.146G > C, c.334C > T, c.335G > A, c.427G > A, c.658C > T, c.659G > C, c.679C > T, c.901C > T, c.902G > A, c.902G > C, c.1024C > T, c.1025G > A, and c.1066C > T. Twenty-three variants were reported as de novo mutational hotspots (Table S3). However, no particular sites responsible for these de novo mutations were identified.

A few cases of homozygous or compound heterozygous female FD patients have been previously reported [30, 37]. However, homozygous or compound heterozygous female FD patients were not identified in the current study or in our previous NBS study [11]. In our NBS study, the frequency of male FD (the allele frequency of FD variants) was estimated to be 1:6212 (0.016%), and the probability of homozygous female was extremely low as 1:38,588,944. Therefore, among female FD patients, only those with heterozygous *GLA* mutations are generally identified. Interestingly, a male patient harboring two *GLA* variants, c.70 T > A /p.W24R and c.1255A > G/p.N419D, was identified in the current study (Table S1). Unfortunately, genetic information regarding his family and his chromosome information were not available. It was unclear whether the two variants were in *cis* on a single X chromosome. Of 100 *GLA* variants, 70 were detected only in single pedigrees, whereas 20 were identified in two pedigrees. Because a bias was introduced in the distribution of variants in these pedigrees, it was difficult to discuss the correlation between genotype and phenotype, especially organ specific pathogenicity.

On follow-up evaluation for each patient, 21 of the 26 novel variants were indicated as pathogenic, namely c.97G > C/p.D33H, c.157A > T/p.N53Y, c.184dupT/p.S62Ffs*18, c.205 T > C/p.F69L, c.207del/p.F69Lfs*52, c.264C > G/p.Y88*, c.329del/p.P110Lfs*11, c.386_389dupTGAA/p.L131Efs*9, c.440G > T/p.G147V, c.563delC/p.Y188Sfs*4, c.610 T > G/p.W204G, c.691_693GAC > TAT/p.D231Y, c.825delC/p.S276Afs*6, c.827G > C/p.S276T, c.848A > G/p.Q283R, c.908 T > C/p.I303T, c.987C > A/p.Y329*, c.1019delG/p.E341Nfs*57, c.1054G > C/p.A352P, c.1085_1088dupCTCG/p.Y365Lfs*11, and c.1100dupT/p.A368Rfs*7. Furthermore, five variants were identified herein, which were not registered in the ClinVar or Fabry-database.org, specifically c.725 T > C/p.I242T, c.801 + 1G > A/p.L268Ifs*3, c.908_923del21/p.S304_310Ldel, c.1124G > A/p.G375E, and c.1165C > G/p.P389A. Patients harboring the aforementioned variants developed FD-related symptoms, and some had even died of stroke or cardiac failure.

Even during high-risk FD screening, individuals are assigned an uncertain diagnosis in the absence of classical FD symptoms and when variants of unknown significance (VOUS) in the *GLA* gene are identified. This leads to a risk of misdiagnosis, inappropriate counseling, and extremely costly treatment. Therefore, numerous studies have attempted to generate a diagnostic algorithm for FD, which maximally excludes these risks [38]. In our high-risk screening, individuals presenting decreased activity (<cutoff levels) with known pathogenic variants, classical signs or symptoms of FD, or a family history of FD were definitely diagnosed with FD. However, among individuals presenting decreased activity (<cutoff levels) with VOUS and late onset signs or symptoms such as cryptogenic stroke, proteinuria, or LVH without classic type signs or symptoms, a definite diagnosis is difficult to achieve. Moreover, because the disease state during late-onset FD is potentially not improved through ERT [39], the therapeutic effect of ERT does not facilitate the diagnosis of FD. Blood Lyso-Gb3 assays and tissue diagnosis in a myocardial or renal biopsy may be sufficient for a definite diagnosis of FD [40]. Moreover, analysis using iPSC technology, such as Gb3 accumulation in iPSC-derived vascular endothelial cells, may lead to a definite diagnosis [41].

High-risk FD screening has a potential for false-positive findings. Figure 2a, b, and c show the histograms of this high-risk screening for all individuals, men, and women, respectively, in Method I. The median α-Gal A activity was 24.47, 24.50, and 24.06 (AgalU) among all individuals, men, and women, respectively. A dotted line indicates the cutoff value: < 12 [AgalU] for men and < 20 [AgalU] for women; 50% of median α-Gal A activity for men and 80% of median α-Gal A activity for women. Heterozygous female patients have an almost normal range of α-Gal A activity, resulting in false-negative findings in screening studies. Linthorst et al. [42] reported that 40% (16/40) of female patients with FD are not identified, considering a cutoff < 50% of the normal control. Although we used a higher cutoff < 80% of median α-Gal A activity, false-negative findings may have been obtained among the female FD patients herein. Additional tests, such as blood Lyso-Gb3 assays [43], hotspot mutation screening [44], or even whole *GLA* gene sequencing, may improve the rate of false-negative results. Most patients with FD in Taiwan harbor variants out of a pool of only 21 pathogenic mutations [45]. Therefore, in regions such as Taiwan, where hotspot mutations can be detected, hotspot mutation screening is effective for high-risk screening among women. In regions such as Japan, where hotspot mutations cannot be detected and several variants are found, hotspot mutation screening is not as effective. Whole *GLA* gene sequencing is difficult to perform among all female patients included in the high-risk screening group because of cost-balance issues. The assay of Lyso-Gb3 in dried blood spots (DBSs) is considered an effective and realistic alternative for high-risk screening among women [43]. We will consider applying the Lyso-Gb3 assay for high-risk screening in future studies.

Figure 2d shows a histogram of α-Gal A activity in an NBS study in Method I [11]. The median α-Gal A activity among neonates was 42.58 (AgalU), which is approximately 2-fold that of the current high-risk screening populations. FD is associated with a significantly reduced life expectancy compared to that of the general population [45]. Although the detailed mechanism for the low α-Gal A activity in adults is unknown, it may be associated with a premature aging process through the dysfunction of blood vessels. Therefore, aging and low α-Gal A activity are closely related.

The cutoff values in the high-risk screening populations were 12 (AgalU) for men and 20 (AgalU) for women, which is representative of the cutoff values for the 0.5 percentile in the NBS population. This is because α-Gal A activity in adults is lower than that of neonates. The current high-risk screening program identified individuals who are considered suitable candidates for migalastat treatment. Some patients were already receiving migalastat treatment. Moreover, gene therapy holds promise in effectively treating various diseases, and the clinical trials for gene therapy for FD are ongoing in Canada and the USA (https://fabrydiseasenews.com/gene-therapy-for-fabry-disease/). In the future, the development of new treatment methods for FD, other than ERT, is expected.

## Conclusions

In the current study, we performed high-risk screening for FD in individuals from all the prefectures in Japan. A total of 18,199 individuals were screened using DBSs, and 101 *GLA* variants, including 26 novel variants, were identified among 236 patients from 143 families. The distribution of variants is diverse for each region of Japan, and de novo mutations in the *GLA* gene were detected in a significant proportion of these variants. Therefore, further novel mutations would likely be identified in the future. Regarding treatment, 33% of the FD patients were identified as suitable candidates for migalastat therapy and 39% of the *GLA* variants were identified as amenable. Therefore, the simple screening protocol using DBSs could be useful in the early diagnosis and selection of appropriate treatments of FD in high-risk and undiagnosed patients with various renal, cardiac, or neurological manifestations. FD screening is essential for individuals presenting with peripheral neuropathy or a family history of FD as both have been identified as strong predictive factors in FD development.

## Methods

### Study design

To identify FD patients by performing high-risk screening in 18,135 individuals, enrolled from October 2006 to March 2019, with renal, cardiac, or neurological manifestations from all the prefectures in Japan; the following study design was implemented. A total of 601 hospitals, from all the prefectures in Japan, participated in this study. From October 2006 to March 2019, the DBSs of 18,135 patients with various cardiac, renal, or neurological manifestations were assessed. Written informed consent was obtained from the patients or their parents (in cases where the patients were not of legal age). The individuals were enrolled in the study if they developed at least one of the following manifestations: (a) renal manifestations, such as proteinuria, chronic kidney disease, diabetic nephropathy, mulberries in the urine, and the need for dialysis; (b) cardiac manifestations, such as LVH detected using electrocardiography or echocardiography; (c) central neurological manifestations, such as parkinsonism, hearing loss, and history of stroke; (d) peripheral neurological manifestations, including limb pain, acroparesthesia, clustered angiokeratoma, cornea verticillata, and hypo-anhidrosis; (e) family history of FD; or (f) other reasons, such as liver failure and unavailable information.

DBS specimens were prepared as reported previously [17]. Briefly, after dropping blood spots onto filter papers (Toyo Roshi Kaisha, Ltd., Tokyo, Japan), the DBSs were dried for at least 4 h at room temperature, sent to Kumamoto University by mail within 1 week of preparation, and if necessary, stored at − 20 °C until use. The high-risk screening for FD using α-Gal A assays with DBSs was performed in two steps. In the first step, individuals with α-Gal A activity under the cutoff value (in Method I: < 12 [AgalU] for males and < 20 [AgalU] for women; and Method II: < 15 [AgalU] for males and < 20 [AgalU] for women) were recalled and their DBSs re-prepared. In the second step, individuals with α-Gal A activity under the cutoff value were assessed clinically, and *GLA* gene sequencing was performed after informed consent was obtained from the patients or their parents (in cases where the patients were not of legal age).

### α-Gal a assay

#### Method I

α-Gal A assays were performed as described previously [11]. Briefly, a single 3.2 mm diameter disk, punched from DBSs, was incubated in a well of a 96-well clear microwell plate (Corning, NY, USA) with 40 μL of McIlvaine buffer (100 mM citrate; 200 mM NaH_2_PO_4_; 36.8:63.2; pH 6.0) and processed for extraction at room temperature for 2 h. Aliquots of 30 μL blood extract were transferred to fresh 96-microwell plates. An aliquot of 100 μL of the reaction mixture (3.5 mM 4-methylumbelliferyl-α-d-galactopyranoside (4MU-αGal); 100 mM citrate; 200 mM K_2_HPO_4_; 100 mM N-acetyl-d-galactosamine) was added to each well of the microwell plates and incubated at 37 °C for 24 h. The reaction was terminated using 150 μL of termination solution (300 mM glycine/NaOH; pH 10.6) immediately after the reaction occurred. The fluorescence intensity, from the 4-methylumbelliferones in the wells, was measured at 450 nm using a fluorescence plate reader (BIO-TEK, Winooski, VT, USA). One unit (1 AgalU) of enzymatic activity was equal to 0.34 pmol of 4MU-αGal cleaved/h per disk.

#### Method II

Method II for multiple assays was developed in collaboration with KM Biologics Co., Ltd. (see details at JP6360848B) and practically implemented from November 2016. Briefly, a single 3.2 mm diameter disk, punched from DBSs, was incubated in a well of a 96-well clear microwell plate (AS ONE Corporation, Osaka, Japan) with 100 μL of 25 mM citrate/potassium phosphate buffer (pH 6.0) containing 5 mM MgCl_2_, 0.5 mM DTT, 0.05% NaN_3_, and 0.1% Triton X-100 for 1 h at room temperature with gentle mixing. A 20-μL aliquot of the extract was then added to 40 μL of the reaction mixture (3.0 mM 4MU-αGal; 100 mM N-acetyl-d-galactosamine in 100 mM citrate/200 mM potassium phosphate buffer; pH 4.4) in a black 96-well microwell plate (Thermo Fisher Scientific Inc., MA, USA). The reaction mixture was incubated at 38 °C for 3 h, and the reaction was terminated with 200 μL of 300 mM glycine/NaOH buffer (pH 10.6) containing 10 mM ethylenediaminetetraacetic acid (EDTA) to measure fluorescence intensity. The residual extract could be used for the assay of acid α-glucosidase (Pompe disease) and glucocerebrosidase (Gaucher disease) activity.

### Sequencing of the *GLA* gene

#### Sanger sequencing

Genomic DNA was extracted from total blood using a Gentra Puregene Blood Kit (Qiagen, Hilden, Germany), or equivalent product, and stored at − 80 °C until use. All seven exons and the flanking intronic sequences of the *GLA* gene were amplified using PCR as described previously [46, 47]. The region of intron 4 was also amplified to evaluate the variant, c.639 + 919G > A [48]. The PCR products were sequenced using an ABI3500xl autosequencer (Applied Biosystems) and analyzed using Sequencher 5.0 (Gene Codes Corporation, Ann Arbor, MI, USA).

#### Next-generation sequencing (NGS)

A high-throughput NGS assay for *GLA gene*was developed in collaboration with KM Biologics Co., Ltd. and practically implemented from September 2017; the protocol is described in our previous report [11]. Briefly, the 13.3-kb region, including *GLA*, was amplified using long-range PCR. Library preparation and sequencing were performed using a Nextera XT Kit (Illumina, San Diego, CA, USA) and MiSeq sequencer (Illumina). After sequencing runs were completed, the data were aligned with those of the human reference genome sequence (NC_000023.10) using MiSeq Reporter software (Illumina). Sequence data analysis, mapping, and variant calling were streamlined using MiSeq Reporter v2 (Illumina). Sequencing reads were visualized using IGV_2.3.10 (Broad Institute). Variants detected in the *GLA* gene by NGS were resequenced using the Sanger method.

### Prediction and statistical tools

#### Significance analysis for the variants

The *GLA* mRNA reference sequence (RefSeq; NM_000169.2) was used in this study, whereby the “A” nucleotide of the ATG codon at nucleotide position 111 of RefSeq constituted + 1 numbering of the cDNA sequence. The ATG codon also represented + 1 for the amino acid numbering as set forth by the α-Gal A preprotein sequence NP_000160.1. Variant nomenclature followed the guidelines established by the Human Genome Variation Society (http://varnomen.hgvs.org/). Public databases, including Fabry-database.org [1] (http://fabry-database.org/, updated on February 15, 2019), and ClinVar [2] (http://www.ncbi.nlm.nih.gov/clinvar) were used to classify each variant. The software PolyPhen-2 [49] (http://genetics.bwh.harvard.edu/pph2) was used for missense mutations to predict the potential impact of an amino acid alteration on α-Gal A function. The amenability of each variant for the pharmacological chaperone migalastat was confirmed using the website of Amicus Therapeutics, Inc. (http://www.galafoldamenabilitytable.jp/reference).

## Supplementary information

**Additional file 1. **(Microsoft PowerPoint Presentation.pptx): **Figure S1.** Histograms of α-Gal A activity in the high-risk population. A. Renal manifestations group (total, *N* = 8004; male, *N* = 4884; and Female, *N* = 2905). B. Cardiac manifestations group (total, *N* = 2410; male, *N* = 1735; and female, *N* = 651). C. Central neurological manifestations (total, *N* = 2593; male, *N* = 1609; and female, *N* = 803). D. Peripheral neurological manifestations group (total, *N* = 316; male, *N* = 181; and female, *N* = 134). E. Family history group (total, *N* = 334; male, *N* = 141; and female, *N* = 190).

**Additional file 2. **(Microsoft PowerPoint Presentation.pptx): **Figure S2.** Geographic distribution of the variants detected from high-risk screening for Fabry disease in Japan.

**Additional file 3: Table S1.** Demographic characteristics of patients identified by high-risk screening for Fabry disease. NA: not available, (#): Potential alteration of splicing predicted by Human Splicing Finder. Amenability; +: amenable, −: not amenable,?: unknown (http://www.galafoldamenabilitytable.jp/reference, access at 2020/01/06). p.F69L/c.207C > A was reported by Umeda et al., Hum. Genome Var. 2 (2015) 15044. Gray box: novel variants

**Additional file 4: Table S2.** Variants detected during high-risk screening for Fabry disease. Fabry-database.org: last updated 2019/02/15 ver.3.2.2. NR: not registered, (#): Potential alteration of splicing predicted by Human Splicing Finder. ** +: amenable, −: not amenable,?: unknown, NA: not available, http://www.galafoldamenabilitytable.jp/reference (access at 2020/01/06). p.F69L/c.207C > A was reported by Umeda et al., Hum. Genome Var. 2 (2015) 15044. Gray box: novel variants

**Additional file 5: Table S3.** De novo mutations detected in patients with Fabry disease.

## Data Availability

The datasets used and/or analyzed during the current study are available from the corresponding author on reasonable request.

## Abbreviations

DBSs: Dried blood spots
DMD: Duchenne muscular dystrophy
ERT: Enzyme replacement therapy
FD: Fabry disease
NBS: Newborn screening
LVH: Left ventricular hypertrophy
NGS: Next-generation sequencing
α-Gal A: α-galactosidase A
4MU-αGal: 4-methylumbelliferyl-α-d-galactopyranoside
