# Supplementary figures and images for: Fabry disease screening in high-risk populations in Japan: a nationwide study
Source: Orphanet J Rare Dis. 2020 Aug 26;15:220. doi: 10.1186/s13023-020-01494-6 (PMC7448968; doi:10.1186/s13023-020-01494-6)

## Slide 1
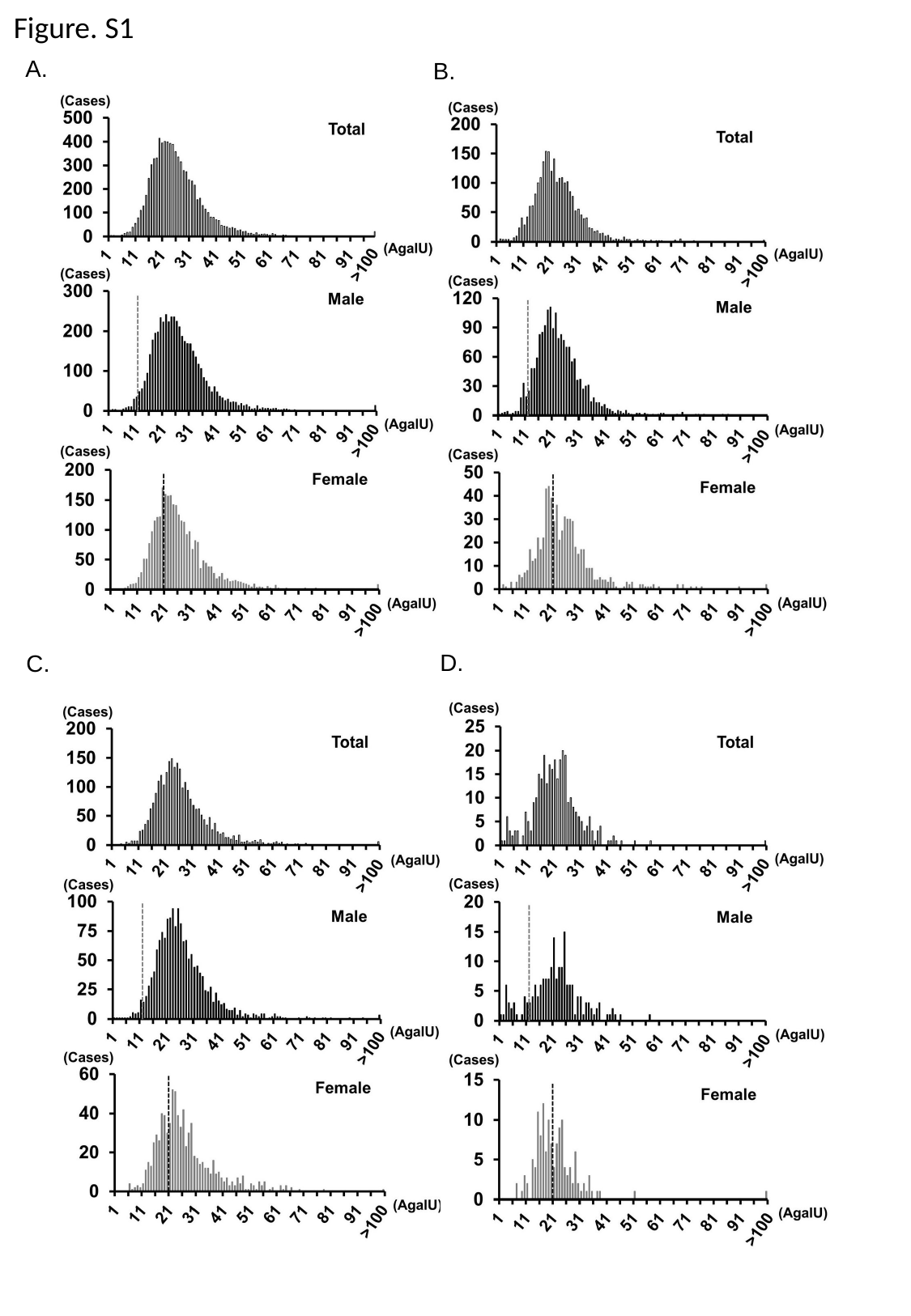

Figure. S1
A.
B.
D.
C.

## Slide 2
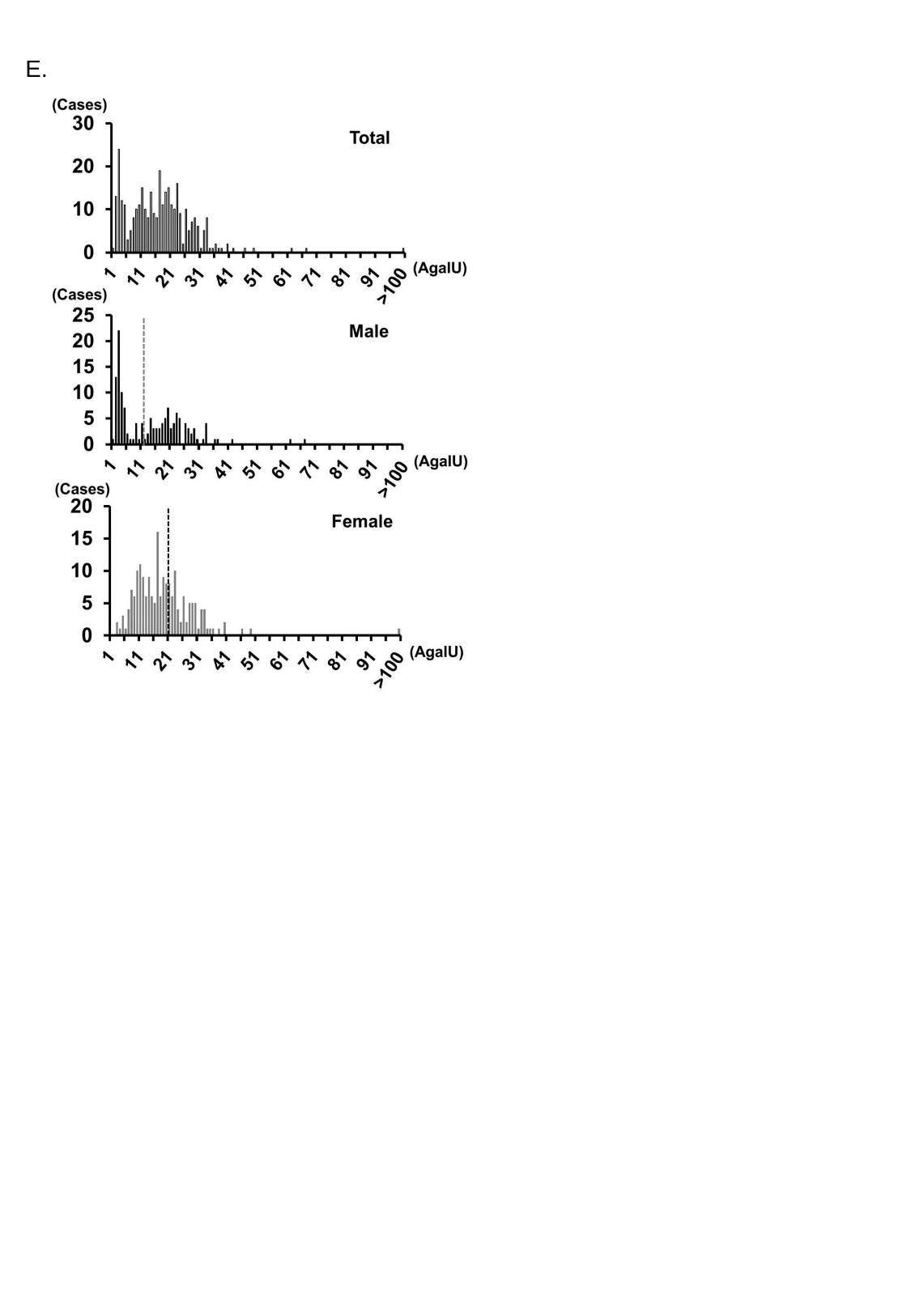

E.

Supplement: Supplementary file 1 — Additional file 1. (Microsoft PowerPoint Presentation.pptx): Figure S1. Histograms of α-Gal A activity in the high-risk population. A. Renal manifestations group (total, N = 8004; male, N = 4884; and Female, N = 2905). B. Cardiac manifestations group (total, N = 2410; male, N = 1735; and female, N = 651). C. Central neurological manifestations (total, N = 2593; male, N = 1609; and female, N = 803). D. Peripheral neurological manifestations group (total, N = 316; male, N = 181; and female, N = 134). E. Family history group (total, N = 334; male, N = 141; and female, N = 190). [file 13023_2020_1494_MOESM1_ESM.pptx]
