# Supplementary material for: Fabry disease screening in high-risk populations in Japan: a nationwide study
Source: Orphanet J Rare Dis. 2020 Aug 26;15:220. doi: 10.1186/s13023-020-01494-6 (PMC7448968; doi:10.1186/s13023-020-01494-6)
Supplement: Supplementary file 2 — Additional file 2. (Microsoft PowerPoint Presentation.pptx): Figure S2. Geographic distribution of the variants detected from high-risk screening for Fabry disease in Japan. [file 13023_2020_1494_MOESM2_ESM.pptx]

## Slide 1
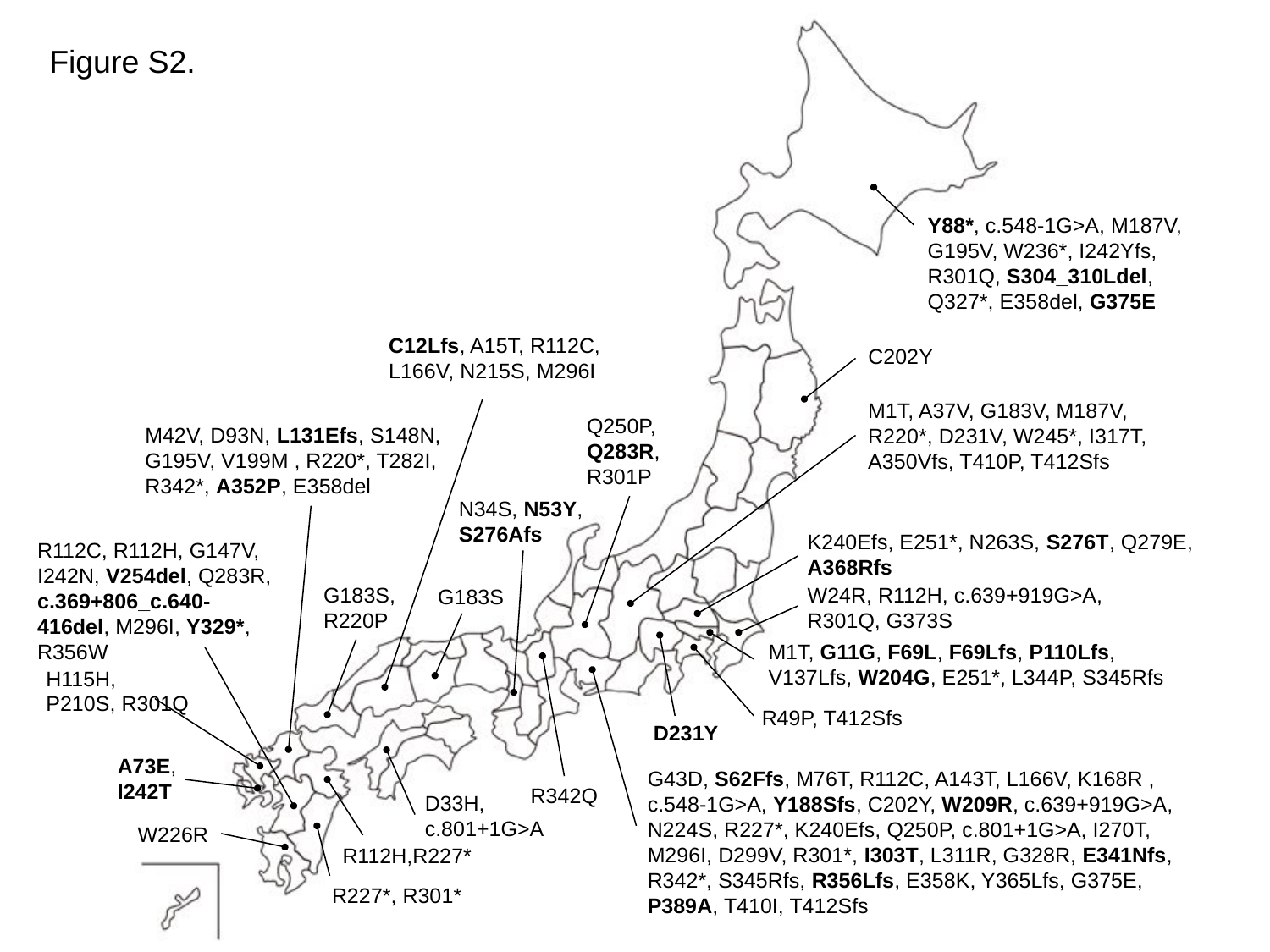

Figure S2.
Y88*, c.548-1G>A, M187V, G195V, W236*, I242Yfs, R301Q, S304_310Ldel, Q327*, E358del, G375E
C12Lfs, A15T, R112C, L166V, N215S, M296I
C202Y
M1T, A37V, G183V, M187V, R220*, D231V, W245*, I317T, A350Vfs, T410P, T412Sfs
Q250P, Q283R, R301P
M42V, D93N, L131Efs, S148N, G195V, V199M , R220*, T282I, R342*, A352P, E358del
N34S, N53Y, S276Afs
K240Efs, E251*, N263S, S276T, Q279E, A368Rfs
R112C, R112H, G147V, I242N, V254del, Q283R, c.369+806_c.640-416del, M296I, Y329*, R356W
W24R, R112H, c.639+919G>A, R301Q, G373S
G183S, R220P
G183S
M1T, G11G, F69L, F69Lfs, P110Lfs, V137Lfs, W204G, E251*, L344P, S345Rfs
H115H, P210S, R301Q
R49P, T412Sfs
D231Y
A73E, I242T
G43D, S62Ffs, M76T, R112C, A143T, L166V, K168R , c.548-1G>A, Y188Sfs, C202Y, W209R, c.639+919G>A, N224S, R227*, K240Efs, Q250P, c.801+1G>A, I270T, M296I, D299V, R301*, I303T, L311R, G328R, E341Nfs, R342*, S345Rfs, R356Lfs, E358K, Y365Lfs, G375E, P389A, T410I, T412Sfs
R342Q
D33H, c.801+1G>A
W226R
R112H,R227*
R227*, R301*
